# Altered mannose metabolism in chronic stress and depression is rapidly reversed by vitamin B12

**DOI:** 10.3389/fnut.2022.981511

**Published:** 2022-10-13

**Authors:** Patricia Franzka, Gustavo Turecki, Susana Cubillos, Takfarinas Kentache, Johann Steiner, Martin Walter, Christian A. Hübner, Olivia Engmann

**Affiliations:** ^1^Institute of Human Genetics, University Hospital Jena, Friedrich Schiller University, Jena, Germany; ^2^Department of Psychiatry, Douglas Mental Health University Institute, McGill University, Montreal, QC, Canada; ^3^Institute for Biochemistry and Biophysics, Friedrich-Schiller-University Jena, Jena, Germany; ^4^Laboratory of Physiological Chemistry, de Duve Institute, Brussels, Belgium; ^5^Clinic for Psychiatry and Psychotherapy, University Hospital of Otto-von-Guericke-Universität Magdeburg, Magdeburg, Germany; ^6^Department of Psychiatry and Psychotherapy, Jena University Hospital, Jena, Germany

**Keywords:** stress, vitamin B12, depression, glycosylation, mannose

## Abstract

GDP-Mannose Pyrophosphorylase B (GMPPB) is a key enzyme for glycosylation. Previous studies suggested a dysregulation of GMPBB and mannose in depression. Evidence, however, was sporadic and interventions to reverse these changes are unknown. Here, we show that GMPPB protein, but not RNA abundance is increased in the postmortem prefrontal cortex (PFC) of depressed patients and the chronic variable stress (CVS) mouse-model. This is accompanied by higher plasma mannose levels. Importantly, a single dose of intraperitoneally administered vitamin B12, which has previously been shown to rapidly reverse behavioral symptoms and molecular signatures of chronic stress in mice, normalized GMPPB plasma mannose levels and elevated GDP-mannose abundance. In summary, these data underline metabolic dysregulation in chronic stress and depression and provide further support for rapid effects of vitamin B12 on chronic stress.

## Introduction

GDP-Mannose Pyrophosphorylase B (GMPPB) is a key enzyme in the glycosylation pathway, which catalyzes the synthesis of GDP-mannose from mannose-1-phosphate and guanosine triphosphate (1). Its activity is regulated by its enzymatically inactive homologue GMPPA, which acts as an allosteric inhibitor of GMPPB. Mutations in GMPPB and GMPPA result in complex congenital disorders of glycosylation (2–4). Recently, a protein-wide association study on depressed patients found that GMPPB protein levels are increased postmortem in the prefrontal cortex (PFC) of depressed patients (5). Accordingly, receptors for neurotransmitters that are essential to stress and depression in the PFC are regulated by glycosylation, including the NMDA receptor (6–8) and serotonin receptors (9, 10). In congenital disorders of glycosylation, depressive symptoms are relatively frequent (11, 12).

The substrate for GMPPB, mannose-1-phosphate, is reduced in the PFC in a rat model of chronic stress and depression (13). A high dose of intraperitoneally administered mannose may contribute to depression-like states in mice (14). However, it is, at least to our knowledge, unknown, whether mannose levels are intrinsically affected by depression and whether this correlates with GMPPB abundance.

We recently observed that vitamin B12 rapidly reverses depression-associated phenotypes in mice (15). Vitamin B12 (cobalamin) is an essential nutrient, which can only be synthesized by bacteria. Vitamin B12 is a cofactor in the one-carbon metabolism, which provides methyl donors, e.g., for the methylation of DNA, histones or other proteins. 15% of the human population suffers from vitamin B12 deficiency and affected individuals have an increased risk of suffering from depression (16–18). Importantly, there is also evidence that vitamin B12-supplementation in non-deficient populations may reduce depression risk (19, 20). We have previously shown that a single acute dose of vitamin B12 reduced depressive-like behavior and stress-linked biomarkers in mice in the chronic mild stress-model (15).

Here we confirm increased GMPPB protein levels in human PFC postmortem tissue of depressed patients. Furthermore, we observed increased mannose levels in plasma of depressed patients. Similar changes were found in the chronic variable stress (CVS) mouse-model, which is superior in modeling human molecular signatures of depression (21). Additionally, plasma GDP-mannose levels were increased by CVS in mice. Interestingly, an acute dose of vitamin B12 was sufficient to reverse GMPPB and plasma mannose levels. In summary, this study underlines an association between GMPPB and depression.

## Methods

### Animals and licenses

Mice were housed in accordance with the ethical guidelines of the Thüringer Landesamt für Verbraucherschutz (TLV). Experiments were conducted under Animal license UKJ-18-037 (Germany), which are complying to the EU Directive 2010/63/EU guidelines for animal experiments. C57Bl/6J mice were bred in the animal facility (FZL) of Jena University Hospital, Germany. Animals received standard chow (LASQCdiet Rod16-R, LASvendi GMBH, Soest, Germany), which contains 50 mg/kg chow vitamin B12. Mice were at least 10 weeks of age. Mice were housed in a 14L:10D light-cycle. For brain analysis, mice were sacrificed and PFC tissue was immediately frozen on dry ice and stored at −80°C until further use.

### Drugs and chemicals

Mice were intraperitoneally (i.p.) injected with 2.7 mg/kg vitamin B12 (cyanocobalamin, #V6629, Sigma-Aldrich, Burlington, MA, USA) or saline at an injection volume of 10 ml/kg body weight and tested 24 h later as described in Engmann et al. (22).

### RNA purification and quantification

RNA was purified by resuspension in Trizol and chloroform-precipitation. RNA was washed in isopropanol and 75% ethanol. After cDNA-conversion with a GoScript™ Reverse Transcriptase kit (#A5001, Promega, Madison, WI, USA), quantitative realtime-PCR was performed on a Bio-rad CFX96 Real-time system. Quantitative PCR results were processed as described (23). Primer sequences were: *GMPPB*/*Gmppb*, Fvd: 5′-CCT CACTGGCATGTGC CTC-3′, Rev: 5′-GACTTGTGGGGC AGCACG-3′; *GAPDH* (22), Fvd: 5′-TGGGCAGCCGTTAGG AAAG-3′, Rev: 5′-AGTTAAAAGCAGCCCTGGTGA-3′; *Gapdh* (22), Fvd: 5′-AACTTTGGCATTGTGGAAGG-3′, Rev: 5′-ACACATTGGGGGTAGGAACA-3′.

### Western blot

Tissue lysates were prepared with the Potter S tissue homogenizer (Sartorius, #S14492) in TBS-buffer (20 mM Tris, 150 mM NaCl, 1% (v/v) TritonX-100, complete protease inhibitor and complete phosphatase inhibitor (#04693124001, Sigma-Aldrich, Burlington, MA, USA). After sonication, homogenates were spun down at 16,900 g to remove nuclei and insoluble debris. The supernatant was stored at −80°C until further use. Proteins were denatured at 90°C for 5 min in Laemmli buffer (4X Laemmli buffer: 50% glycerol, 5% SDS, 0.25%1.5M Tris pH 6.8, 30% β-mercaptoethanol, 0.001% bromophenol blue, ddH_2_O). After separation by SDS-PAGE (8% polyacrylamide glycine gels, run for 1.5 h at 80 V) proteins were transferred onto 0.45 μm PVDF membranes (#10600023, GE Healthcare) at 290 mA for 100 min. Membranes were blocked in 2% BSA for 1 h at RT and incubated with primary antibodies at appropriate dilutions in tris-buffered saline supplemented with 0.1% Tween-20 (TBS-T) overnight at 4°C. The following primary antibodies were used: rabbit anti-GMPPA (#15517-1-AP, Proteintech, Rosemont, IL, USA) 1:500, rabbit anti-GMPPB (#15094-1-AP, Proteintech, Rosemont, IL, USA) 1:500, rabbit anti-GAPDH (#10494-1-AP, Proteintech, Rosemont, IL, USA) 1:1,000, self-made mouse anti-oligomannose antibody (6–9 terminal mannose residues) 1:50 [gift of Rüdiger Horstkorte, Halle (24)]. Membranes were washed in TBS-T and primary antibodies were detected with horseradish peroxidase-conjugated secondary antibodies in an appropriate dilution. Following secondary antibodies were used: donkey anti-rabbit IgG-HRP (#NA 9340V, Amersham, Buckinghamshire, UK) 1:4,000 and goat anti-mouse IgM # (31440, Thermo Fisher Scientific, Waltham, MA, USA) 1:4,000.

### Measurement of plasma sugar concentrations

Human plasma samples were obtained in agreement with the Ethics committee of Magdeburg University Hospital, Germany (110/07). Patients did not receive psychopharmacological substances other than benzodiazepines for at least 6 weeks prior to testing. Blood plasma was collected by centrifuging blood immediately after collection for 10 min at 3,000 rpm (Hettich centrifuge EBA 2, type 2002). The supernatant consisting of plasma was aliquoted into DNA LoBind tubes (Eppendorf, Hamburg, Germany) and stored at −80°C. Samples from depressed patients and age-matched controls were collected at day 0 (T0) and 6 weeks after the beginning of treatment (T6). Treatment differed between patients and was individually tailored to their needs. As no difference was found in plasma sugar levels for patients between days T0 and T6 (data not shown), the average of both time points was calculated for each participant to reduce variability. Mouse blood was obtained from unfasted animals and incubated on ice for 15 min. Samples were centrifuged for 10 min at 4°C and 4,000 g. Sugars were measured in the supernatant with the D-mannose, D-fructose, D-glucose kit following manufacturer’s instructions (#K-MANGL, Megazyme, Wicklow, Ireland).

### GDP-mannose measurements

Plasma samples were obtained as described above and stored at −80°C. For sample preparations, 10 μL of plasma was added to 190 μL of Methanol/water/Chloroform mixture (8:1:1), followed by a vigorous shacking and then centrifuged at 13,200 rpm during 15 min at 4°C. Supernatant was dried by speed Vacuum and samples were re-suspended in 70 μL of methanol 50%, and finally were analyzed by LC/MS as described previously (2). Tissue quality control showed no differences in GMP, GDP, or GTP levels between treatments (data not shown).

### Chronic variable stress induction

The CVS-protocol was performed as described (21). In brief, mice received 21 days of stress with one of three stressors presented in a semi-random order, where the same stressor does not occur on two consecutive days. The following stressors were used: 1 h of tube restraint, tail suspension or 100 mild electric random foot shocks. If only female experimenters were present, a used male t-shirt was wrapped in clean protective clothing from the animal unit and placed into the experimental room in order to avoid variability due to sex-specific scents of the scientists (3). All experiments were conducted in the light phase of the light-cycle to allow comparability with previous experiments (15). For CVS-groups, vitamin B12 was injected at the last day of CVS (day 21), just prior to the stressor.

### Postmortem brain samples

Samples were generously provided by the Douglas-Bell Canada Brain Bank. Experiments were conducted in agreement with the Ethics committee of Jena University Hospital, Germany (Reg.-Nr. 2020-1862-Material) and Douglas Institute REB Approval #04/21.

### Statistics

Statistical analysis was performed in GraphPrism. Two-tailed Student’s *t*-test was used for comparison of two groups. Two-way ANOVA with Bonferroni *post-hoc* test was used, when two factors were varied. Outliers were removed when data points were more than two standard deviations away from the average.

## Results

### GDP-mannose pyrophosphorylase B and plasma mannose are increased in depressed patients and in a mouse model of chronic stress

**FIGURE 1:**
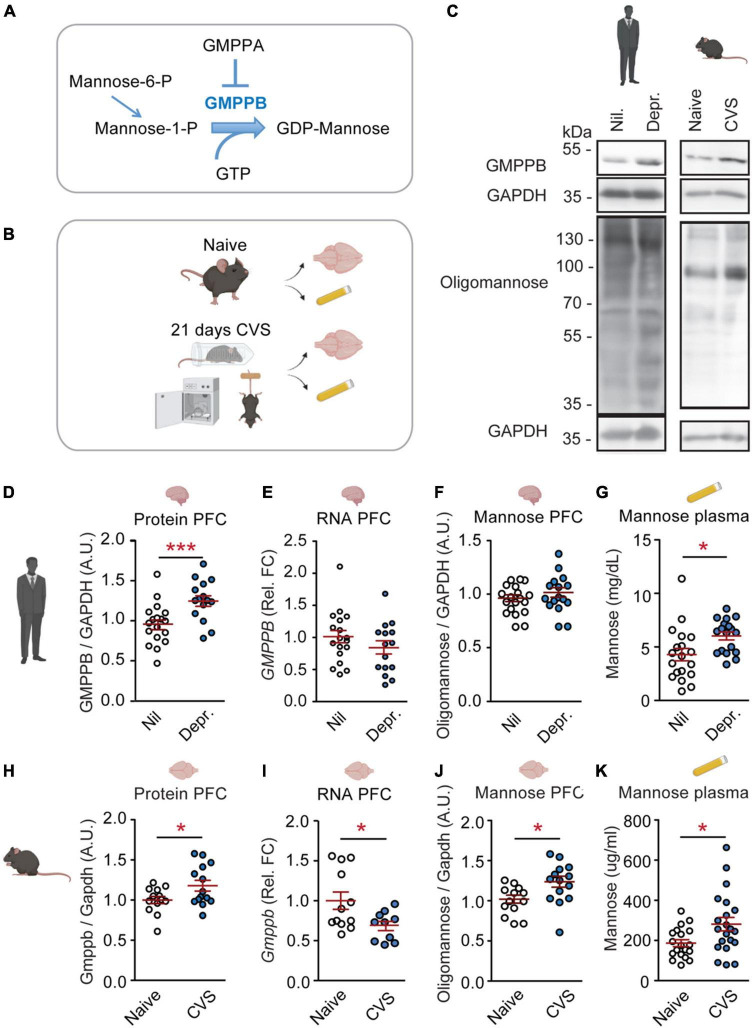
GMPPB and plasma mannose are increased by chronic stress and depression. **(A)** Cartoon illustrating the regulation of GDP-mannose production. **(B)** Experimental setup for CVS-experiment. **(C)** Representative western blots. **(D–K)** Statistics: Student’s *t*-test. **(D–G)** Results from human cohorts. **(D–F,H–J)** Prefrontal cortex. **(G,K)** Plasma. **(D)** Increased GMPPB protein-levels in depressed patients (Depr.) vs. controls (Nil); *n* = 15–18 per group; t_31_ = 3.32, **P < 0.01. **(E)** No association between depression and *GMPPB* RNA-levels; *n* = 15–18 per group; t_31_ = 1.22, *P* = 0.23. **(F)** No association between depression and oligomannose-levels. *n* = 15–19 per group; t_32_ = 0.59, *P* = 0.56. **(G)** Increased plasma mannose in depressed patients; *n* = 18–19 per group; t_35_ = 2.51, *P < 0.05. **(H–K)** Results from the CVS mouse-model. **(H)** Increased GMPPB protein-levels in stressed mice; *n* = 13–14 per group; t_25_ = 2.18, *P < 0.05. **(I)** Reduced *Gmppb*-RNA levels in CVS-group; *n* = 10–12 per group; t_20_ = 2.13, *P < 0.05. **(J)** Increased oligomannose-levels in stressed mice; *n* = 13–14 per group; t_25_ = 2.53, *P < 0.05. **(K)** Increased plasma mannose in CVS-group. *n* = 19–21 per group; t_38_ = 2.44, *P < 0.05. **(D–K)** Individual data points are plotted and means ± s.e.m. are shown. A.U., Arbitrary units; Rel. FC, Relative fold change. Illustrations were generated with biorender.com.

We obtained PFC postmortem tissue from depressed patients to assess whether altered GMPPB levels observed by Wingo et al. (5) can be reproduced and whether this was associated with altered mannosylation (Figure 1A). Additionally, we obtained samples from the CVS mouse-model (Figure 1B). This model is the gold standard in mimicking molecular and behavioral changes linked to depression and is well suited for functional and interventional studies (21, 25).

Increased GMPPB protein levels in postmortem PFC-tissue from depressed patients were confirmed in our cohort (Figures 1C,D). The change in GMPPB protein abundance was not accompanied by increased *GMPPB* transcripts, suggesting a regulation of the protein itself (Figure 1E). As GMPPB is a mediator of mannosylation, oligomannose levels in the PFC and plasma mannose levels were measured as well. While PFC oligomannose levels were not affected in depressed patients (Figure 1F), plasma mannose levels were increased (Figure 1G and Supplementary Figure 1).

In the CVS mouse-model, increased GMPPB was observed as well (Figure 1H). Here, too, *Gmppb* RNA-levels did not match the observed increase in protein abundance (Figure 1I). In the mouse model, both, PFC oligomannose and plasma mannose were increased (Figures 1J,K).

These data suggest that chronic stress and depression are indeed linked to altered PFC GMPPB and mannose metabolism. GMPPB and oligomannose changes in the CVS-model occurred in the PFC. No changes were observed in the hippocampus (Supplementary Figure 2). Moreover, GDP-mannose levels were significantly increased in murine plasma samples (Supplementary Figure 3). Mannose can be converted from other carbohydrates such as fructose and glucose (Supplementary Figure 4). Hence, plasma fructose and glucose levels were assessed in mice and human cohorts as well. While fructose was not affected in depressed patients or by CVS, glucose amounts were significantly reduced in depressed patients (Supplementary Figure 4).

### Vitamin B12 rapidly reverses GDP-mannose pyrophosphorylase B- and plasma mannose-levels in chronically stressed mice

Previously, we have observed that a single dose of vitamin B12 at the end of a chronic stress paradigm can ameliorate symptoms associated with depression (15).

**FIGURE 2:**
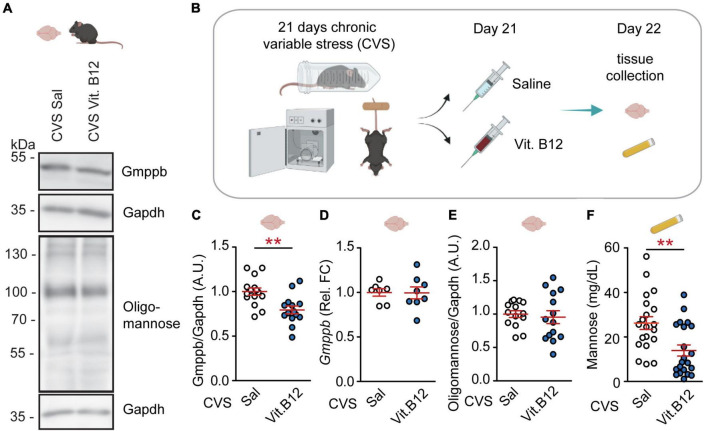
Vitamin B12 rapidly reverses GMPPB levels and plasma mannose in stressed mice. **(A)** Representative western blots. **(B)** Experimental setup for Vitamin B12-experiment. **(C–F)** Statistics: Student’s *t*-test. **(C)** Vitamin B12 reduces GMPPB protein-levels in the PFC of stressed mice; *n* = 14–15 per group; t_27_ = 3.43, **P < 0.01. **(D)** No effect of vitamin B12 on *Gmppb* transcription; *n* = 7–8 per group; t_13_ = 0.05, *P* = 0.96. **(E)** No effect of vitamin B12 on oligomannose abundance in the PFC; *n* = 14 per group; t_26_ = 0.42, *P* = 0.68. **(F)** Vitamin B12 reduces plasma mannose in CVS-treated mice; *n* = 20–21 per group; t_39_ = 3.23, **P < 0.01. **(C–F)** Individual data points are plotted and means ± s.e.m. are shown. A.U., Arbitrary units. Illustrations were generated with biorender.com.

We observed that an acute dose of vitamin B12 reduced GMPPB protein-amounts in stressed mice (Figures 2A–C). *Gmppb* RNA-levels and PFC oligomannose were not affected (Figures 2D,E). Additionally, vitamin B12 decreased plasma mannose levels (Figure 2F) without affecting plasma fructose and glucose (Supplementary Figure 4). Moreover, plasma GDP-mannose levels were not altered by vitamin B12 (Supplementary Figure 3). These data suggest that in mice, a single dose of vitamin B12 can, at least in part, counteract chronic stress-induced changes in mannose metabolism.

### GMPPA, an allosteric inhibitor of GDP-mannose pyrophosphorylase B, is affected by depression, but not chronic stress or vitamin B12

**FIGURE 3:**
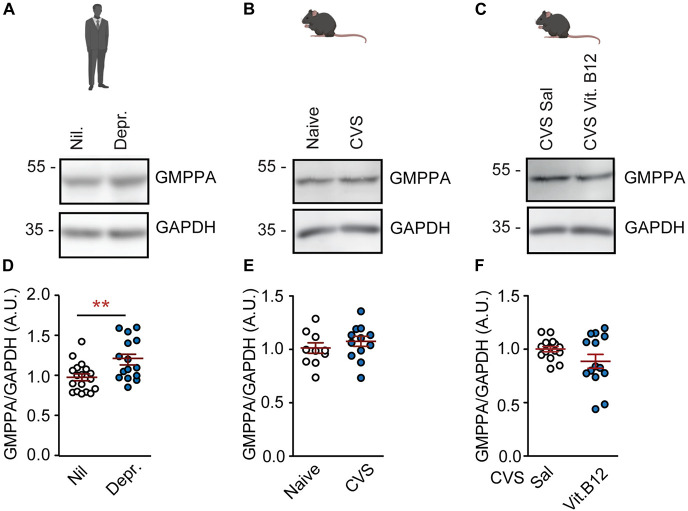
GMPPA levels are not associated with depression or chronic stress in the PFC. **(A)** Representative western blots for GMPPA and housekeeping gene, GAPDH on prefrontal cortex tissue. **(A)** Human cohorts. **(B)** Naïve mice and CVS model. **(C)** Vitamin B12 treatment in stressed mice. **(D–F)** Statistics: Student’s *t*-test. **(D)** Increased GMPPA abundance in depression; *n* = 15–18 per group; t_31_ = 2.91, **P < 0.01. **(E)** GMPPA is not altered by chronic stress; *n* = 13–15 per group; t_26_ = 1.10, *P* = 0.28. **(F)** Vitamin B12 does not affect GMPPA levels; *n* = 13–15 per group; t_26_ = 1.95, *P* = 0.06. **(D–F)** Individual data points are plotted and means ± s.e.m. are shown. A.U., Arbitrary units. Illustrations were generated with biorender.com.

GMPPA is the allosteric feedback inhibitor of GMPPB (Figure 1A). GMPPA protein levels were increased in depressed patients (Figures 3A,D). However, they were not affected by chronic stress in untreated mice or by vitamin B12 in stressed mice (Figures 3B–F and Supplementary Figure 5). Hence, the observed GMPPB-changes appear to be selective in mice. In human depression cohorts, GMPPB-associated elevations in mannosylation might be counteracted by an increase in GMPPA abundance as a compensatory mechanism.

## Discussion

Here we confirm a previous observation that altered GMPPB levels are increased in the postmortem PFC of depressed patients. We found similar changes in a mouse model of chronic stress and depression. Changes in mice were specific to the PFC and were not accompanied by altered GMPPA-levels. Notably, in a brain proteome-wide association study, Wingo et al. found not only GMPPB levels to be altered, but also beta 3-glucosyltransferase (B3GALTL). This indicates that various enzymes in the glycosylation process might be changed upon depression. We further observed increased plasma mannose-levels in patients suffering from depression. Unfortunately, it was not possible to obtain correlative data as the samples had to be taken from different alive and postmortem cohorts. Furthermore, we found that vitamin B12 can rapidly decrease GMPPB and plasma mannose in stressed mice.

While the CVS-model reflected main observations from human depressed patients, it differed in several details. For instance, *Gmppb* transcript-levels were reduced in stressed mice but not in depressed patients. Stressed mice, but not depressed patients, showed altered protein-bound oligo-mannose residues in the PFC. Plasma glucose-levels were decreased in depressed patients but not in stressed mice. In humans, but not mice, GMPPA levels were altered. These differences need to be considered when exploring functional links between GMPPB metabolism and depression. Causes may be species-differences or secondary biases due to life style-changes in depressed patients or sampling methods (e.g., altered dietary choices, fasting prior to testing in humans vs. mice, longer postmortem intervals in human samples). Furthermore, depressed patients were under medication (benzodiazepines for plasma cohort, various pharmaceuticals for the postmortem cohort), which may affect metabolic markers as well.

Based on the availability of samples, our data sets were skewed toward males and Caucasians. In order to ensure a wider applicability of findings, such biases should be avoided whenever possible in the future.

The discrepancy between regulation of *Gmppb* transcripts vs. proteins suggest a regulation on a protein level, e.g., via posttranslational modifications or proteasomal degradation. Furthermore, a clear link between vitamin B12 and carbohydrate mechanisms has not been explored. Being a regulator of methyl donors, it is conceivable that vitamin B12 affects the methylation of GMPPB or its’ regulators, for instance on it’s R357 residue (26). This possibility should be further investigated.

Currently it is unclear whether increased GMPPB abundance in the PFC will lead to increased plasma mannose levels, e.g., via degradation of glycoproteins. It is conceivable that, despite the observed brain-region specific alterations, GMPPB abundance may be altered in other tissues such as liver (27). We recently showed that hyperglycosylation of proteins is correlated with increased plasma mannose levels (2). Moreover, we showed that increased GMPPB levels correlate with protein hypermannosylation and enhanced plasma mannose levels (24). Altered glycosylation may affect protein stability and conformation, protein interactions and adhesion, as well as protein activity and localization (28).

In depressed patients and CVS mice, we found plasma mannose concentrations to be strongly increased, which may reflect increased release from glycans or increased generation of mannose from glucose or fructose. Although only approximately 2% of mannose entering the cell is used for glycosylation (29), the higher systemic mannose levels may contribute to the larger pool of GDP-mannose/hypermannosylated proteins in CVS mice and patients and thus hyperglycosylation.

In agreement with this, we detected increased plasma GDP-mannose levels in stressed mice. Plasma GDP-mannose levels are probably derived from blood cells or dead peripheral cells and not from an efflux from cells as reported for blood mannose (30). Plasma GDP-mannose levels were not affected by vitamin B12 in stressed mice. This may be due to previously observed tissue-specific effects of vitamin B12. Future studies might address blood glycoproteins and their regulation through vitamin B12 as well.

It has been shown that reduced GMPPB abundance leads to decreased GDP-mannose levels and thus affects neuronal and muscle development (4, 31, 32). For example, motor neurons were shortened (4), and an early marker of pan-neuronal cells was remarkably decreased in GMPPB knockdown zebrafish (33). Notably, GDP-mannose supplementation restored GDP-mannose levels, protein mannosylation and thus muscle and neuronal defects (4). Another study showed that GDP-fucose supplementation in a GDP-mannose 4,6 dehydratase mutant zebrafish where fucosylation was decreased restored protein fucosylation and the neuronal phenotype (34). However, it has been shown that extremely elevated GDP-mannose levels affect neuron morphology and development (4). Thus, normal neuron function likely depends on a balanced GDP-mannose homeostatis mediated by GMPPB and GMPPA.

A possible link between GMPPB and plasma mannose levels may be addressed using GMPPB mutant mice. Furthermore, the impact of a mannose-enriched or mannose-depleted diet on symptoms of chronic stress and depression could be investigated.

This study further supports the rapid stress-ameliorating effects of vitamin B12. The current findings add a metabolic and possible protein-regulatory level to the previously observed changes in behavioral and transcriptional markers, highlighting a multidimensional impact of vitamin B12. To date, the vitamin B12 induced stress reversal has been observed in two different mouse models of chronic stress.

Hence, vitamin B12 should be tested as a rapid dietary intervention to treat symptoms associated with chronic stress and depression in human cohorts. Blood samples may be taken to investigate an impact on plasma mannose in addition to mood-related measures. This would allow in patient-correlations and may perhaps provide a therapeutic approach using a widely available, affordable, well-tolerated and rapid acting molecule to improve symptoms of stress and depression.

Despite the missing functional link between vitamin B12 and mannose metabolism, this study provides several novel insights: (1) Systemic mannose levels are altered in depressed patients and in a mouse model. The fact that vitamin B12 rapidly reverses plasma mannose changes in stressed mice demonstrates the dynamic nature of this marker. (2) Vitamin B12 rapidly normalizes metabolic correlates of depression in a mouse model. Together with previous behavioral and molecular data, this study further encourages testing of vitamin B12 as a fast-acting intervention to chronic stress.

## Data availability statement

The raw data supporting the conclusions of this article will be made available by the authors, without undue reservation. Raw data western blot images are shown in Supplementary Figure 5.

## Ethics statement

The studies involving human participants were reviewed and approved by the 2020-1862-Material. The patients/participants provided their written informed consent to participate in this study. The animal study was reviewed and approved by the UKJ-18-037. Human plasma samples were obtained in agreement with the Ethics committee of Magdeburg University Hospital, Germany (110/07).

## Author contributions

OE provided the study design and ideas, conducted chronic-stress and RNA-experiments, and wrote the manuscript. PF performed the western blots and sugar measurements in plasma and performed the statistical analyses. SC performed the qPCR. GT and JS provided the human samples. TK analyzed the GDP-mannose levels. CH and MW provided the financial support and mentorship. All authors contributed to the article and approved the submitted version.

## Supplementary material

Supplementary Figure 1 Demography of human cohorts. **(A)** Postmortem prefrontal cortex tissue. **(B)** Plasma samples. **(C)** Plasma-mannose levels (from Figure 1) analyzed by sex; *n* = 9 per group; 2-way ANOVA: effect of depr.: *F*(1, 32) = 13.68, *P* = 0.0008; effect of sex: *F*(1, 32) = 0.02, *P* = 0.88; no interaction: *F*(1, 32) = 2.14, *P* = 0.15; Bonferroni *post hoc* test: effect of depr. within females: ^**^*P* < 0.01; all other comparisons: *P* > 0.05. **(D)** Legend with abbreviations. **(C)** Individual data points are plotted and means **±** s.e.m. are shown. Illustrations were generated with biorender.com.

Supplementary Figure 2 Hippocampal GMPPB and oligomannose are not affected by chronic stress or depression. **(A)** Representative western blots on hippocampal tissue. **(B–D)** Statistics: Student’s *t*-test. **(B)** No difference in GMPPB-levels; *n* = 9 per group; t_16_ = 1.10, *P* = 0.29. **(C)** No difference in GMPPA-levels; *n* = 10 per group; t_18_ = 0.69, *P* = 0.50. **(D)** No difference in oligomannose-levels; *n* = 9–10 per group; t_17_ = 0.18, *P* = 0.86. **(B–D)** Individual data points are plotted and means **±** s.e.m. are shown. A.U., Arbitrary units. Illustrations were generated with biorender.com.

Supplementary Figure 3 GDP-Mannose is increased in plasma of chronically stressed mice but not altered by vitamin B12. **(A,B)** Statistics: Student’s *t*-test. **(A)** GDP-mannose is affected by CVS. *n* = 9–10 per group; t_17_ = 2.95, ^**^*P* < 0.01. **(A)** GDP-mannose is not affected by vitamin B12 in the CVS group. *n* = 9–10 per group; t_17_ = 1.21, *P* = 0.24. Individual data points are plotted and means **±** s.e.m. are shown. A.U., Arbitrary units.

Supplementary Figure 4 Fructose and glucose levels in chronic stress and depression. **(A)** Overview of conversion pathway between mannose, fructose and glucose. **(B–G)** Statistics: Student’s *t*-test. **(B,C)** Plasma sugar levels in human cohorts. **(B)** Fructose-levels are not significantly altered in depressed patients; *n* = 19–20 per group; t_37_ = 1.86, *P* = 0.07. **(C)** Glucose levels are reduced in depressed patients. *n* = 19 per group; t_36_ = 3.80, ^***^*P* < 0.001. **(D,E)** Plasma sugar levels in mice. **(D)** Fructose levels are not affected by CVS; *n* = 19–20 per group; t_37_ = 1.15, *P* = 0.26. **(E)** Glucose levels are not altered by CVS; *n* = 19–21 per group; t_38_ = 1.47, *P* = 0.15. **(F)** Vitamin B12 does not affect plasma fructose levels; *n* = 20–21 per group; t_39_ = 0.13, *P* = 0.90. **(G)** Plasma glucose levels are not affected by vitamin B12 in stressed mice; *n* = 20 per group; t_38_ = 1.96, *P* = 0.06. **(B–G)** Individual data points are plotted and means **±** s.e.m. are shown. Illustrations were generated with biorender.com.

Supplementary Figure 5 Full length western blots.
