# Supplementary figures and images for: Altered mannose metabolism in chronic stress and depression is rapidly reversed by vitamin B12
Source: Front Nutr. 2022 Oct 13;9:981511. doi: 10.3389/fnut.2022.981511 (PMC9609420; doi:10.3389/fnut.2022.981511)

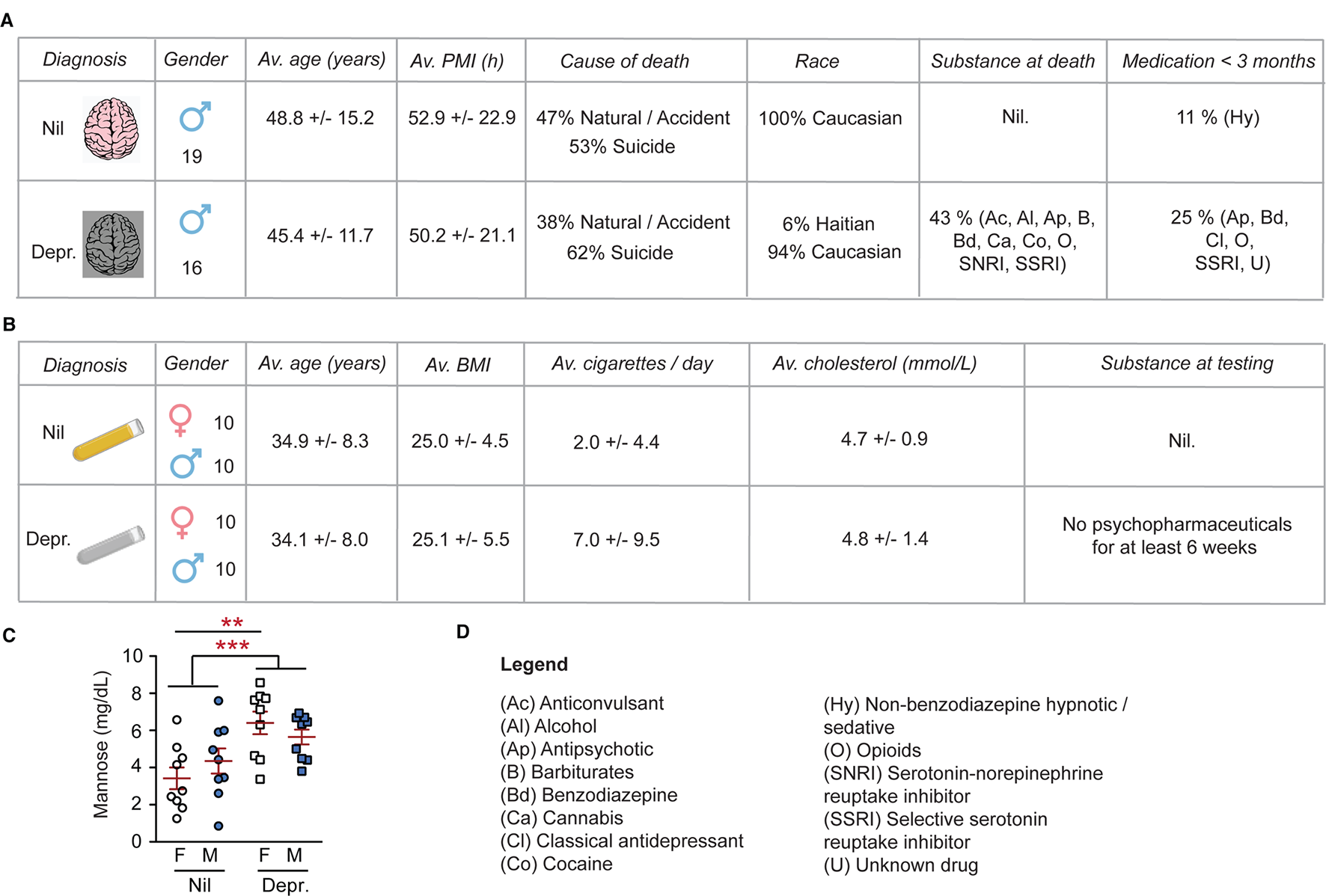

Supplement: Supplementary Figure 1 — Demography of human cohorts. (A) Postmortem prefrontal cortex tissue. (B) Plasma samples. (C) Plasma-mannose levels (from Figure 1) analyzed by sex; n = 9 per group; 2-way ANOVA: effect of depr.: F(1, 32) = 13.68, P = 0.0008; effect of sex: F(1, 32) = 0.02, P = 0.88; no interaction: F(1, 32) = 2.14, P = 0.15; Bonferroni post hoc test: effect of depr. within females: **P < 0.01; all other comparisons: P > 0.05. (D) Legend with abbreviations. (C) Individual data points are plotted and means ± s.e.m. are shown. Illustrations were generated with biorender.com. [file Image_1.TIF]

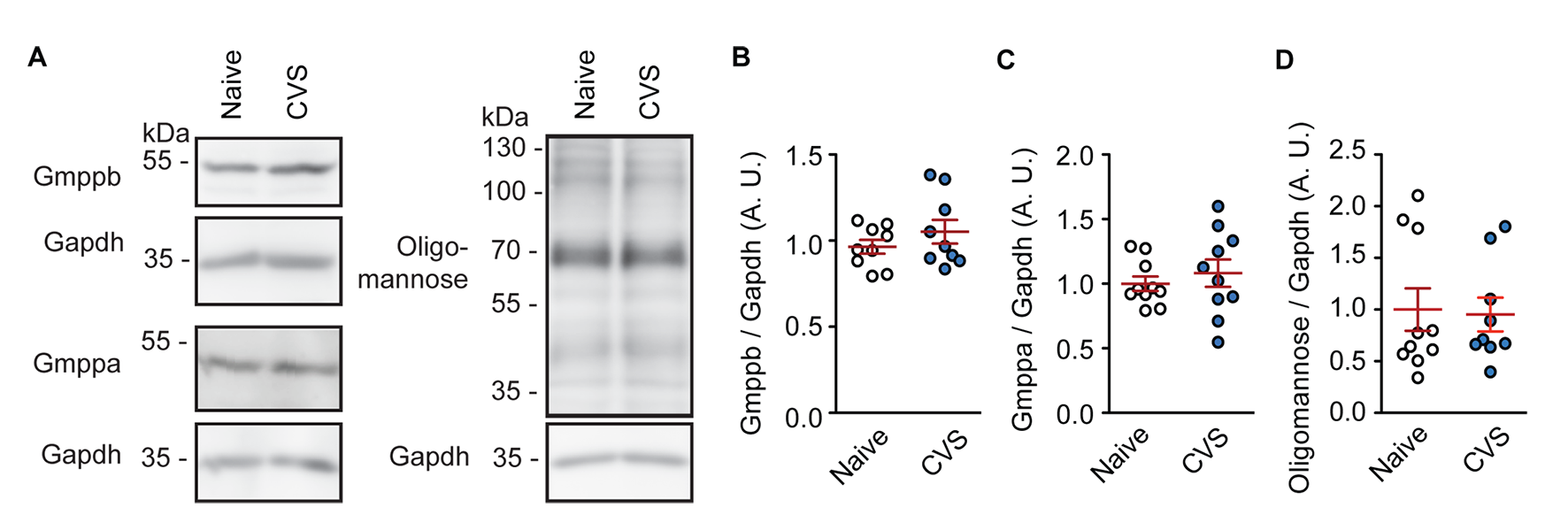

Supplement: Supplementary Figure 2 — Hippocampal GMPPB and oligomannose are not affected by chronic stress or depression. (A) Representative western blots on hippocampal tissue. (B–D) Statistics: Student’s t-test. (B) No difference in GMPPB-levels; n = 9 per group; t16 = 1.10, P = 0.29. (C) No difference in GMPPA-levels; n = 10 per group; t18 = 0.69, P = 0.50. (D) No difference in oligomannose-levels; n = 9–10 per group; t17 = 0.18, P = 0.86. (B–D) Individual data points are plotted and means ± s.e.m. are shown. A.U., Arbitrary units. Illustrations were generated with biorender.com. [file Image_2.tif]

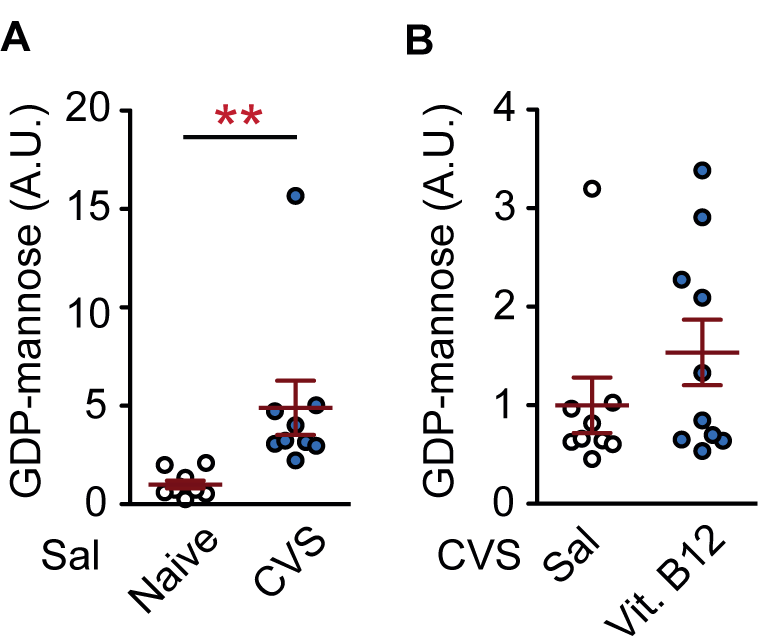

Supplement: Supplementary Figure 3 — GDP-Mannose is increased in plasma of chronically stressed mice but not altered by vitamin B12. (A,B) Statistics: Student’s t-test. (A) GDP-mannose is affected by CVS. n = 9–10 per group; t17 = 2.95, **P < 0.01. (A) GDP-mannose is not affected by vitamin B12 in the CVS group. n = 9–10 per group; t17 = 1.21, P = 0.24. Individual data points are plotted and means ± s.e.m. are shown. A.U., Arbitrary units. [file Image_3.TIF]

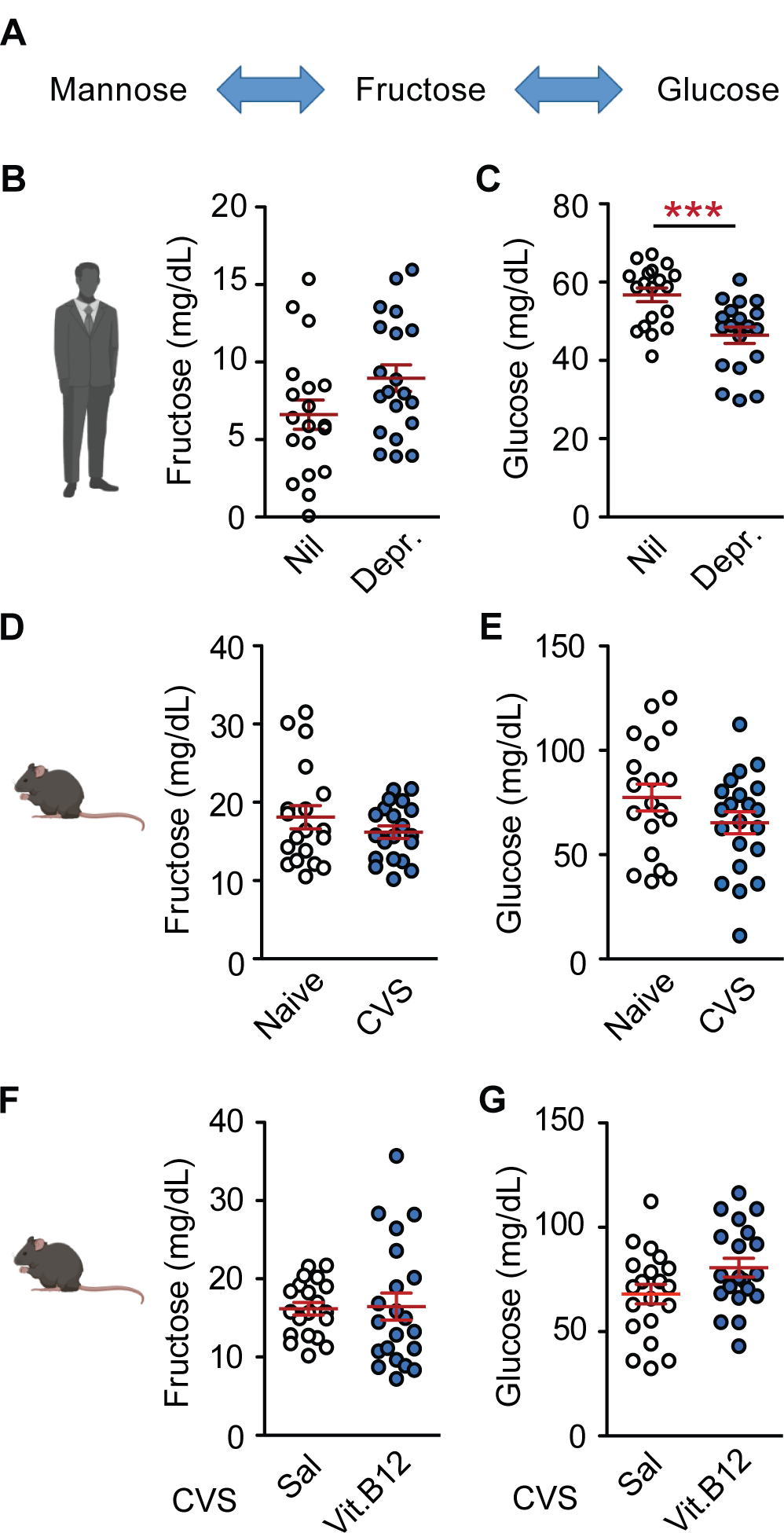

Supplement: Supplementary Figure 4 — Fructose and glucose levels in chronic stress and depression. (A) Overview of conversion pathway between mannose, fructose and glucose. (B–G) Statistics: Student’s t-test. (B,C) Plasma sugar levels in human cohorts. (B) Fructose-levels are not significantly altered in depressed patients; n = 19–20 per group; t37 = 1.86, P = 0.07. (C) Glucose levels are reduced in depressed patients. n = 19 per group; t36 = 3.80, ***P < 0.001. (D,E) Plasma sugar levels in mice. (D) Fructose levels are not affected by CVS; n = 19–20 per group; t37 = 1.15, P = 0.26. (E) Glucose levels are not altered by CVS; n = 19–21 per group; t38 = 1.47, P = 0.15. (F) Vitamin B12 does not affect plasma fructose levels; n = 20–21 per group; t39 = 0.13, P = 0.90. (G) Plasma glucose levels are not affected by vitamin B12 in stressed mice; n = 20 per group; t38 = 1.96, P = 0.06. (B–G) Individual data points are plotted and means ± s.e.m. are shown. Illustrations were generated with biorender.com. [file Image_4.TIF]
