# Supplementary material for: Altered mannose metabolism in chronic stress and depression is rapidly reversed by vitamin B12
Source: Front Nutr. 2022 Oct 13;9:981511. doi: 10.3389/fnut.2022.981511 (PMC9609420; doi:10.3389/fnut.2022.981511)

GMPPB

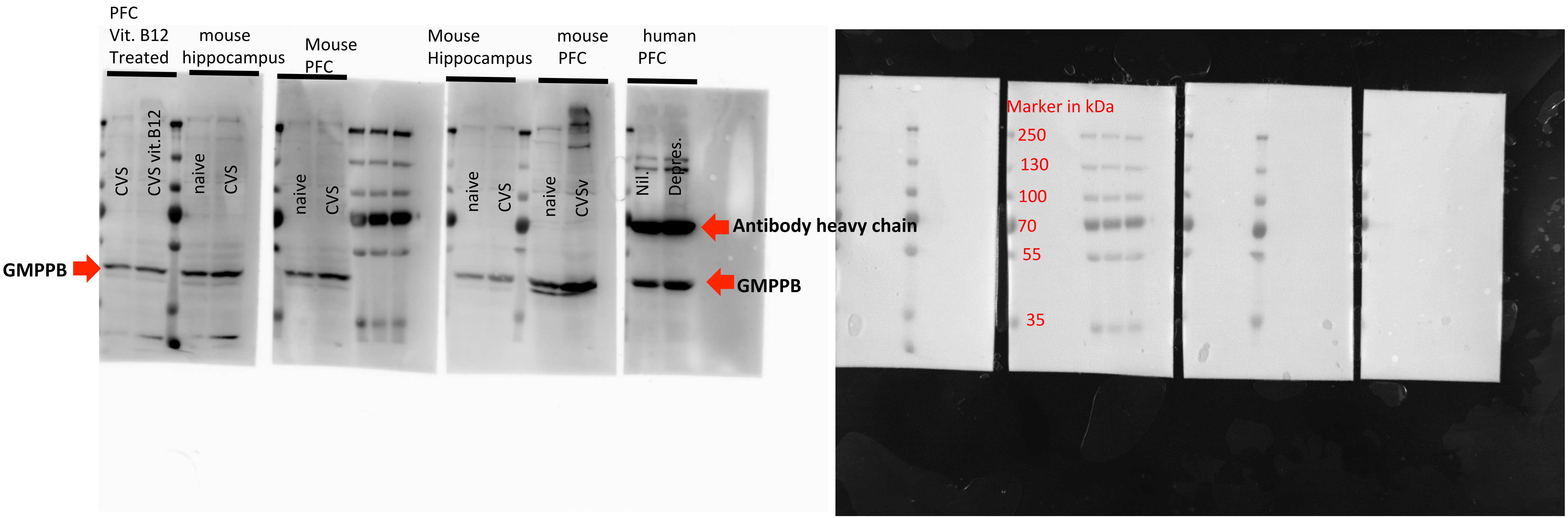
PFC Vit. B12 Treated
mouse hippocampus
Mouse PFC
Mouse Hippocampus
mouse PFC
human PFC
CVS
CVS vit.B12
naive
CVS
naive
CVS
naive
CVS
naive
CVSv
Nil.
Depres.
GMPPB
Antibody heavy chain
GMPPB
Marker in kDa
250
130
100
70
55
35

GAPDH (as loading control for GMPPB)

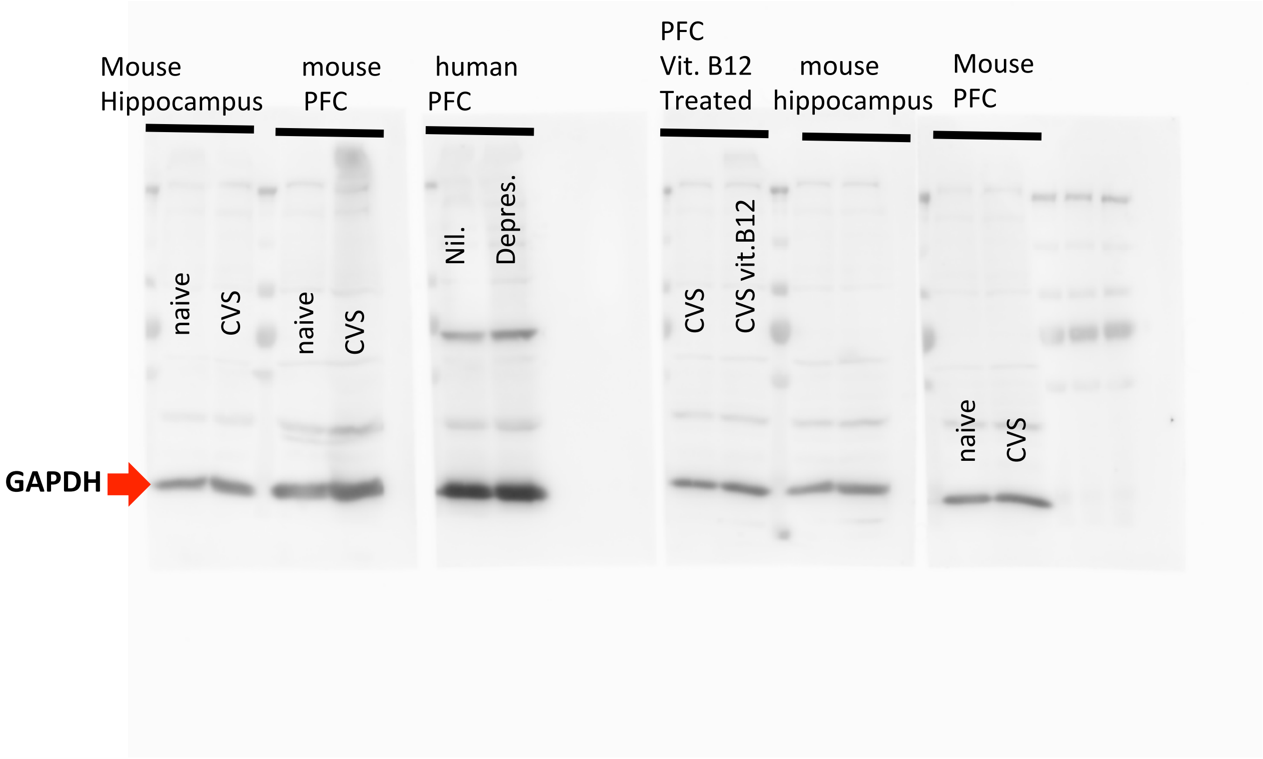

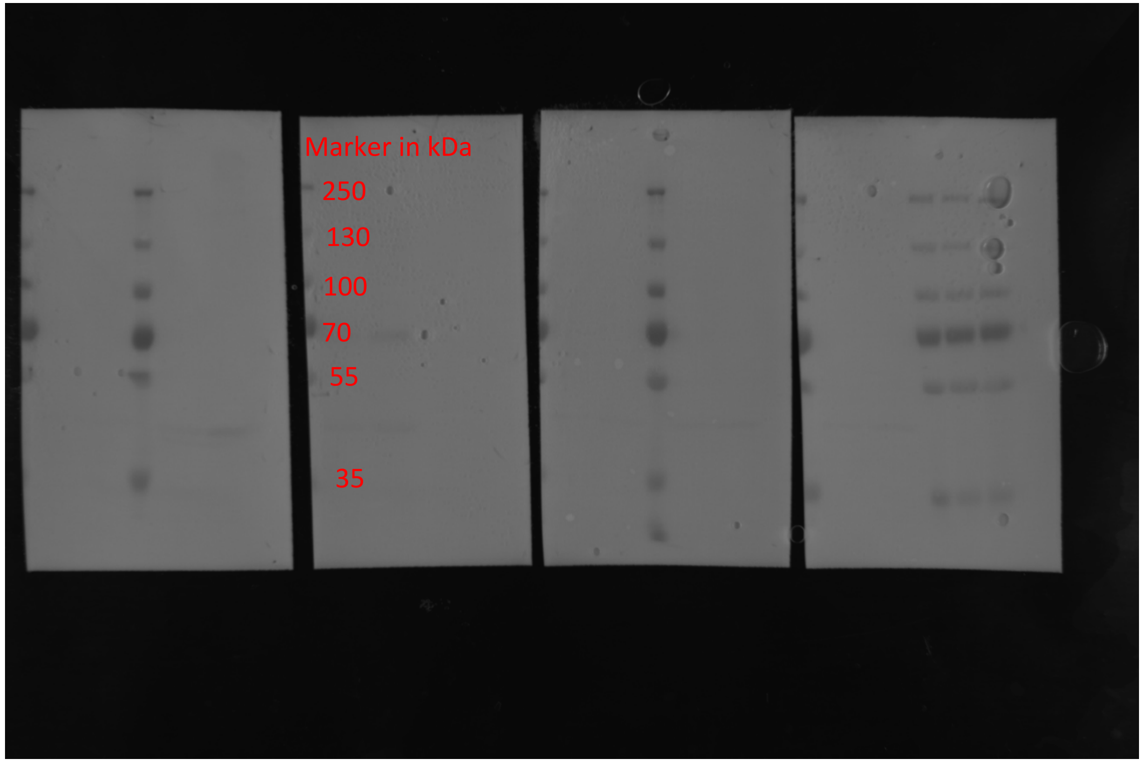

GMPPA

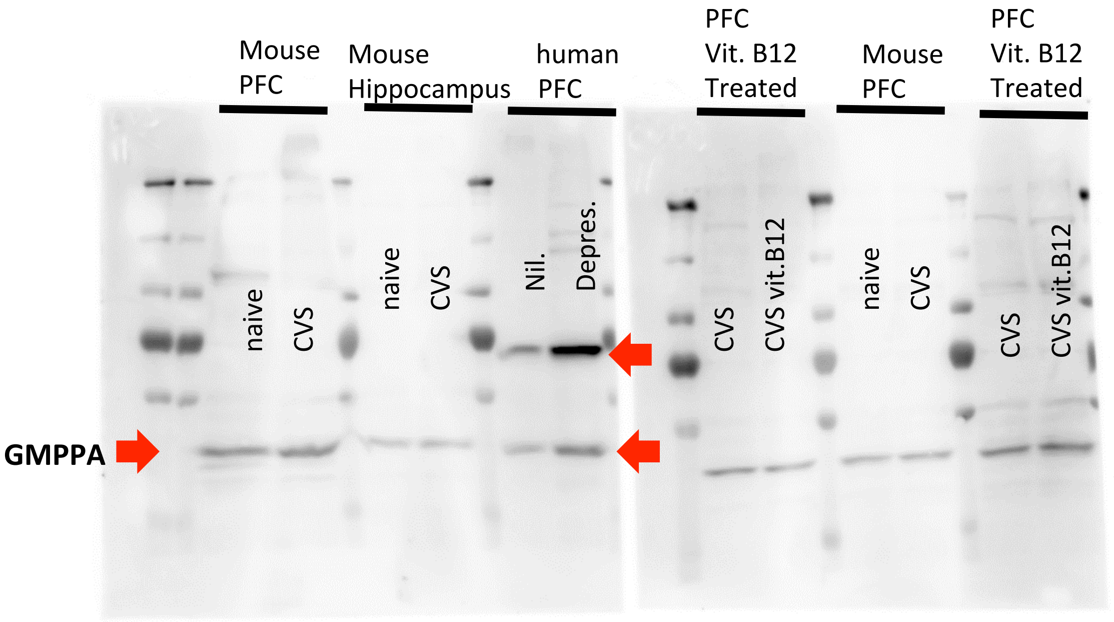
Mouse PFC
Mouse Hippocampus
human PFC
PFC Vit. B12 Treated
Mouse PFC
PFC Vit. B12 Treated
naive
CVS
naive
CVS
Nil.
Depres.
CVS
CVS vit.B12
naive
CVS
CVS
CVS vit.B12
GMPPA

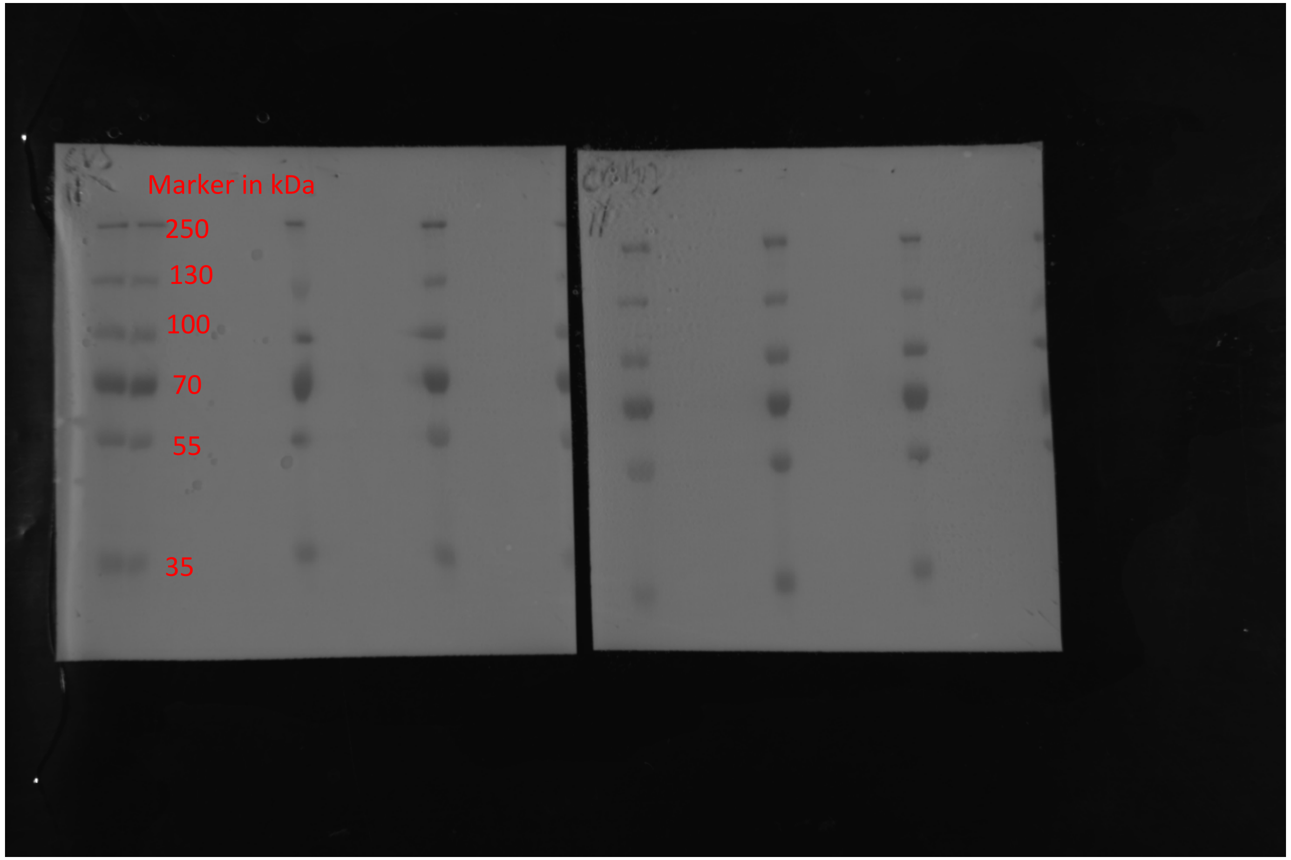
Marker in kDa
250
130
100
70
55
35

GAPDH (as loading control for GMPPA)

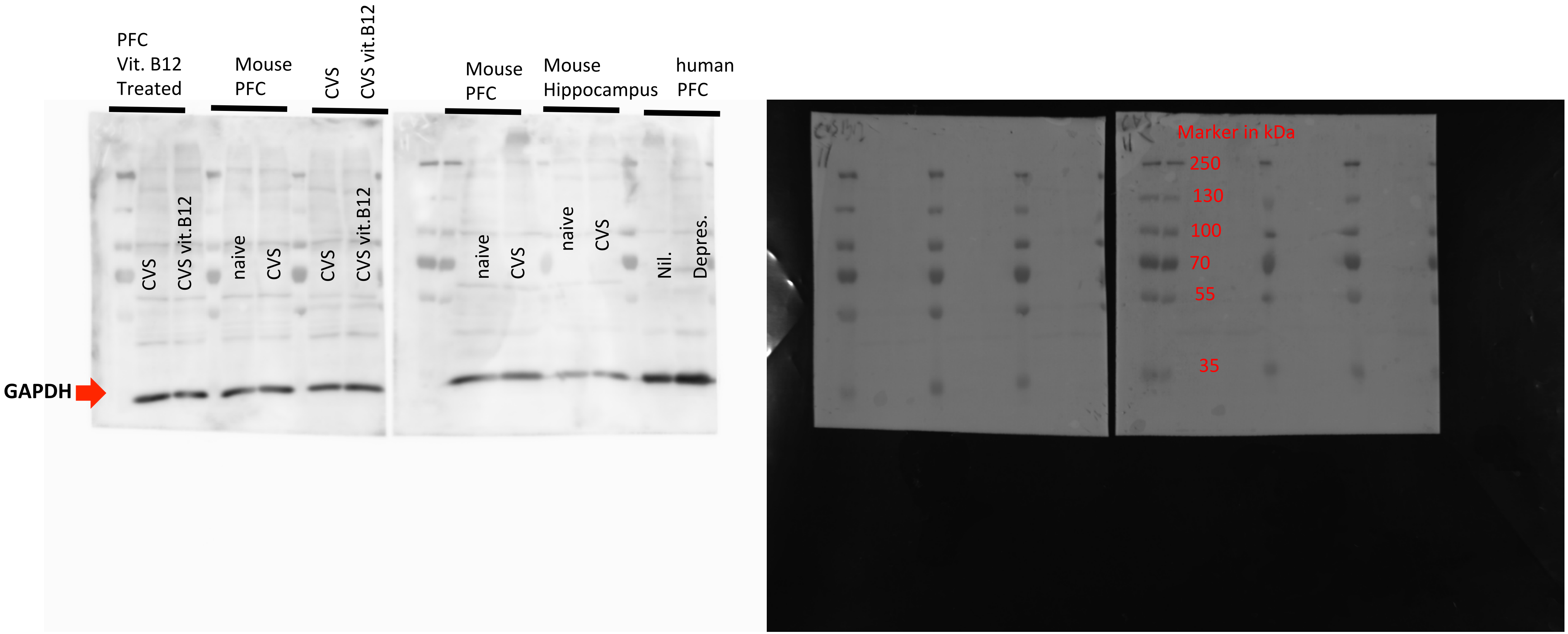

mannosylation

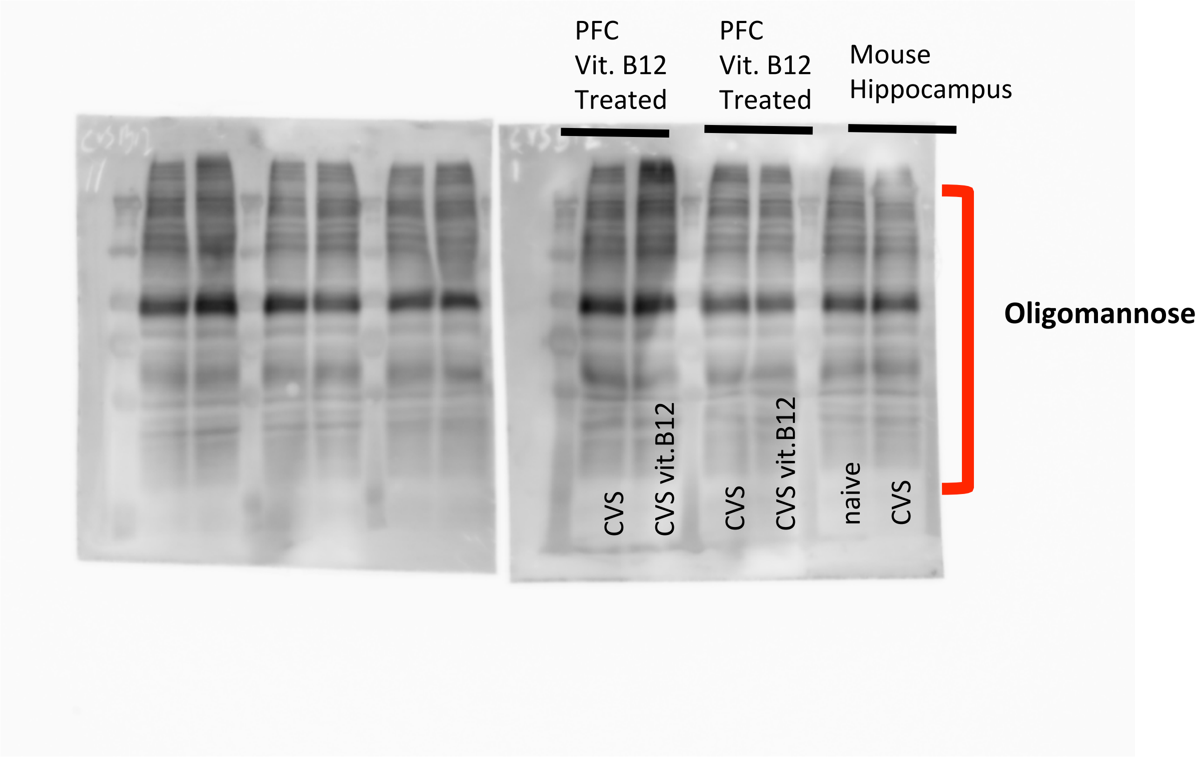

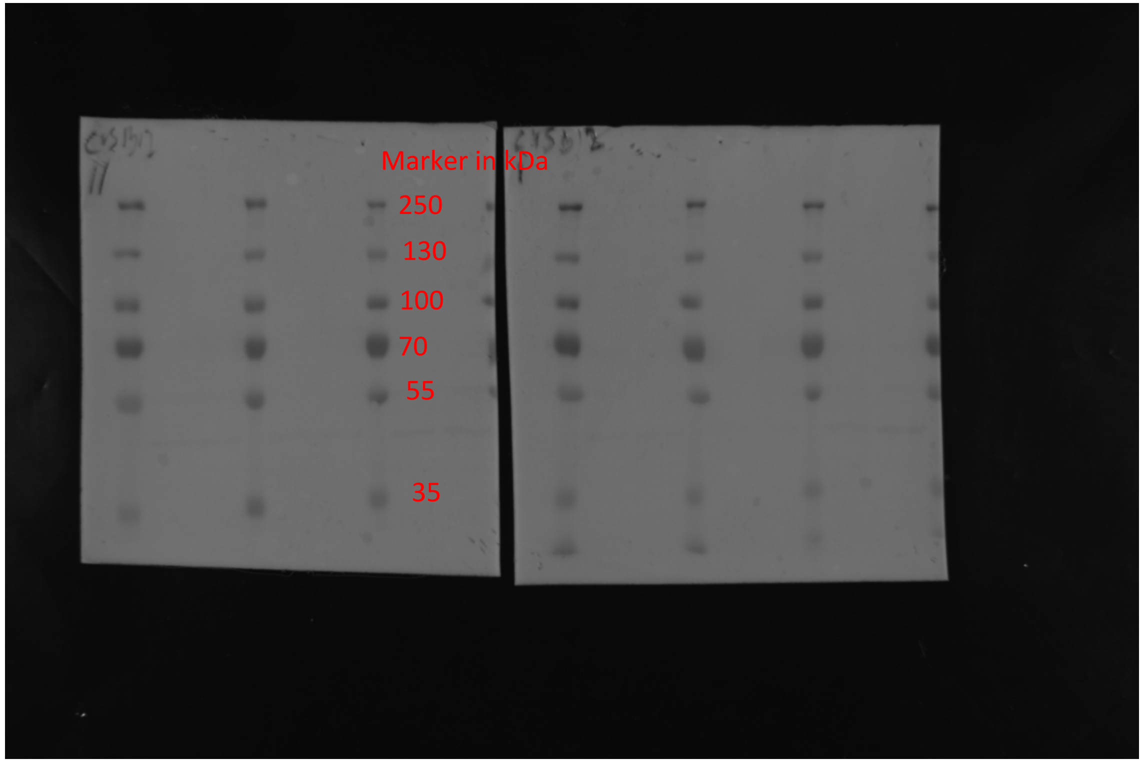

mannosylation

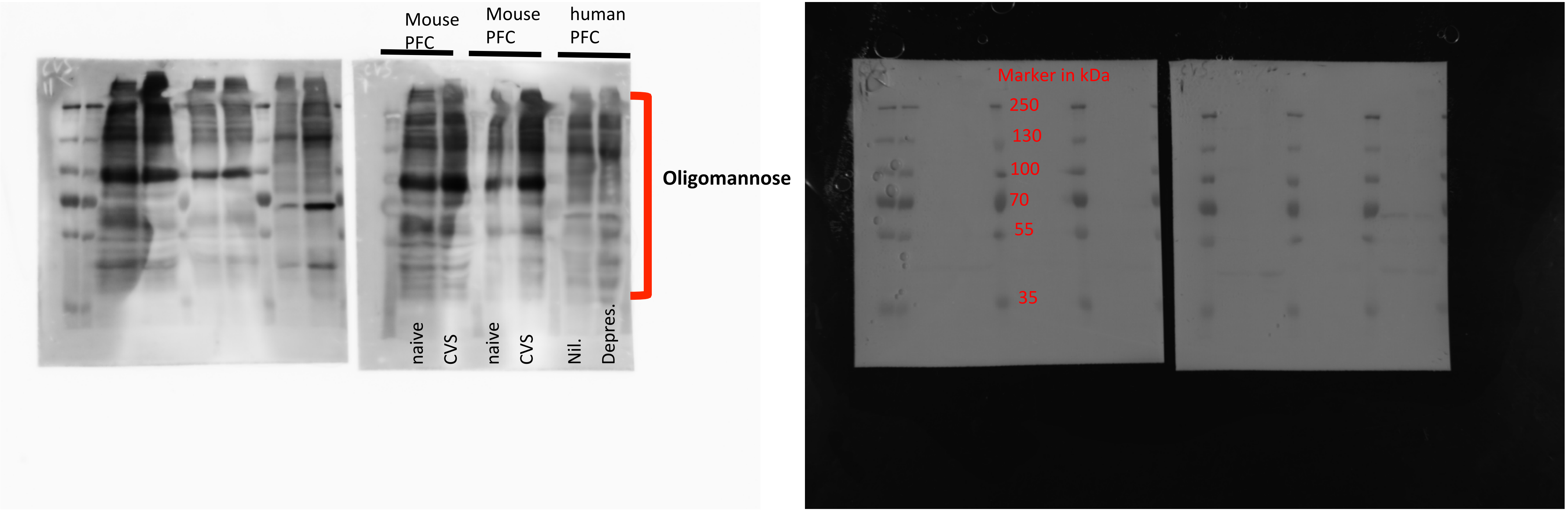

GAPDH (as loading control for mannosylation)

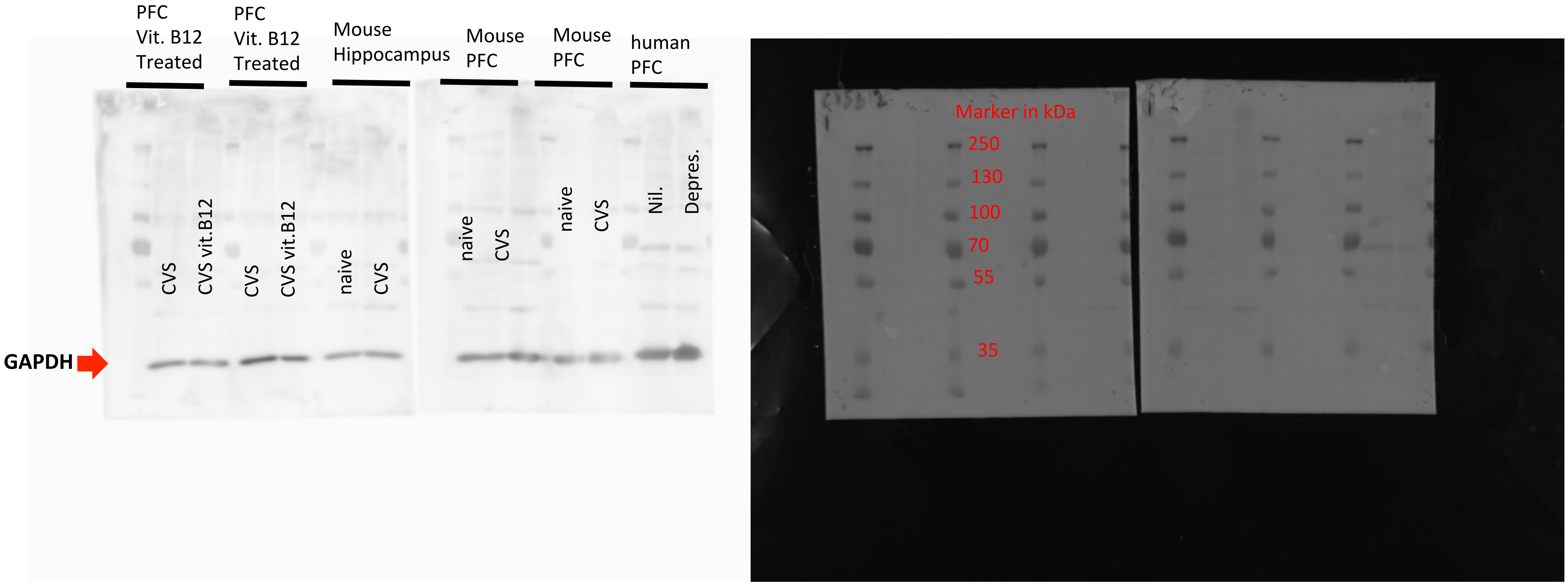

GMPPB (on membranes for mannosylation (before incubated with Oligomannose antibody)

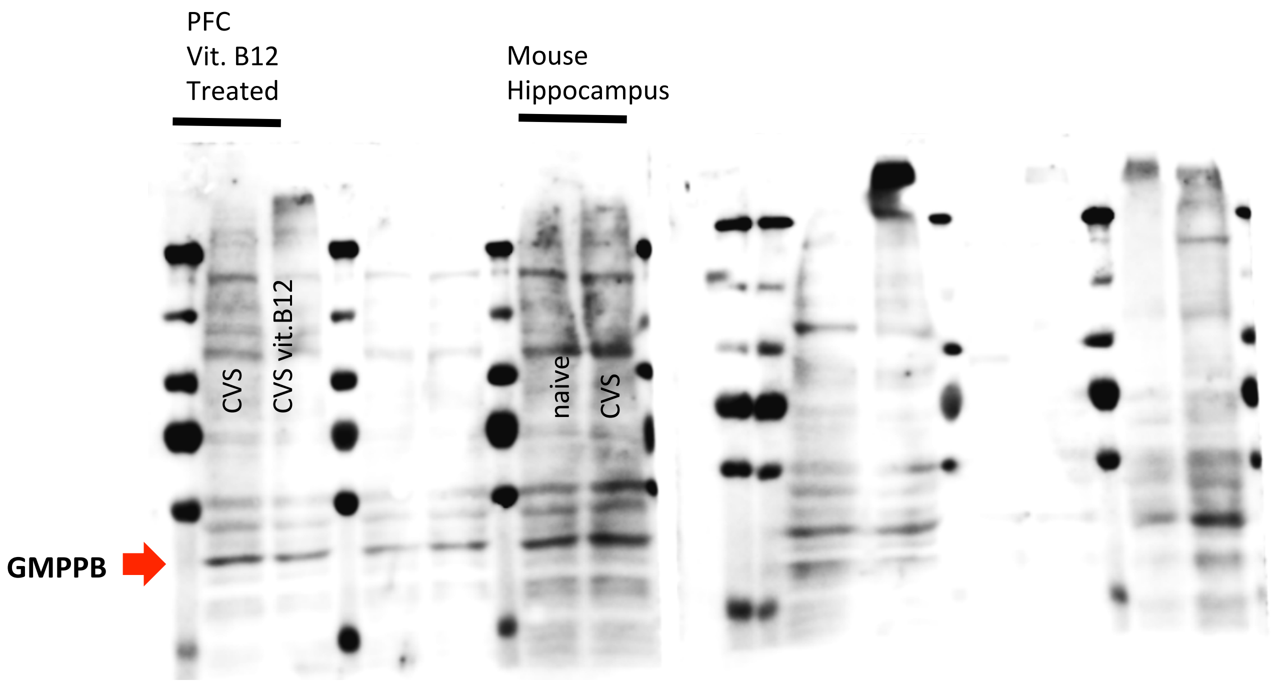

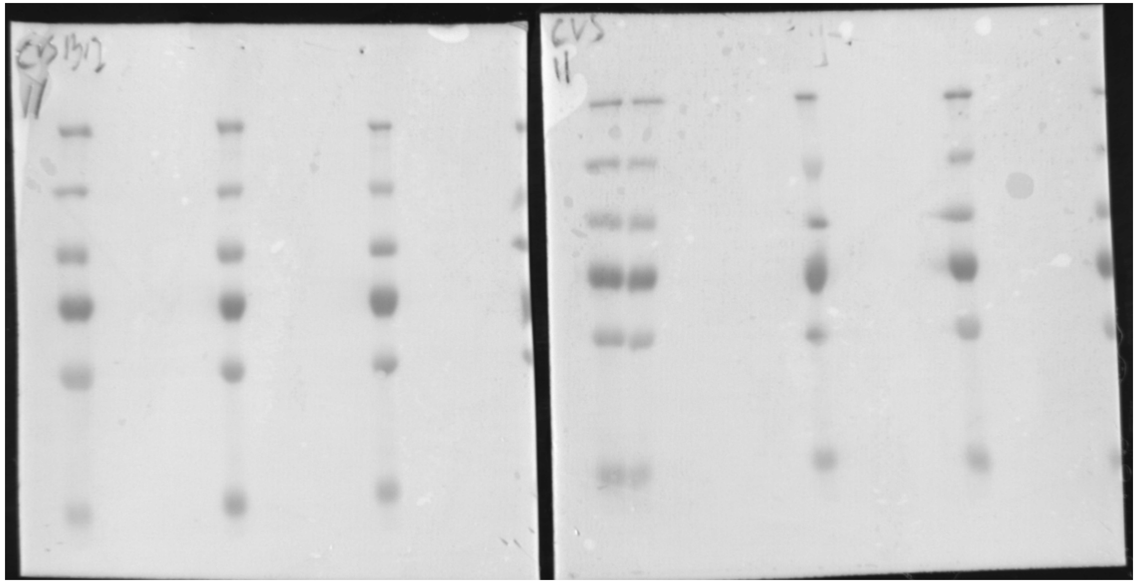

Coomassie staining (as total protein control)

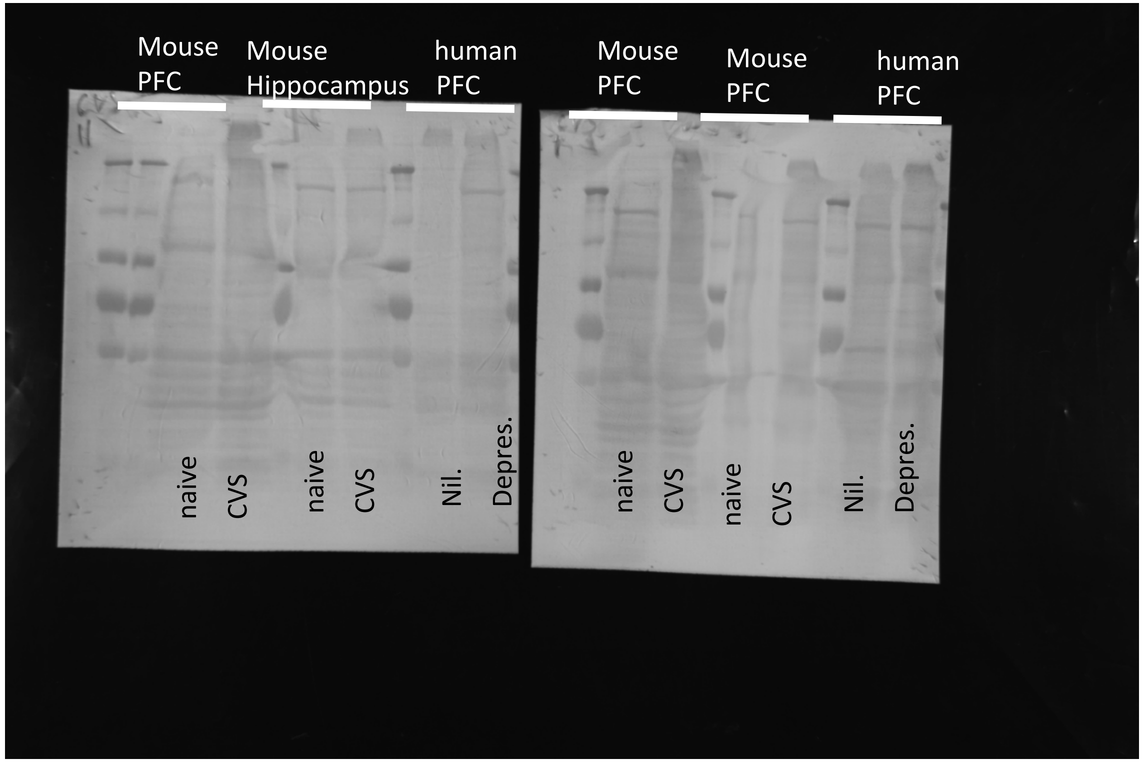

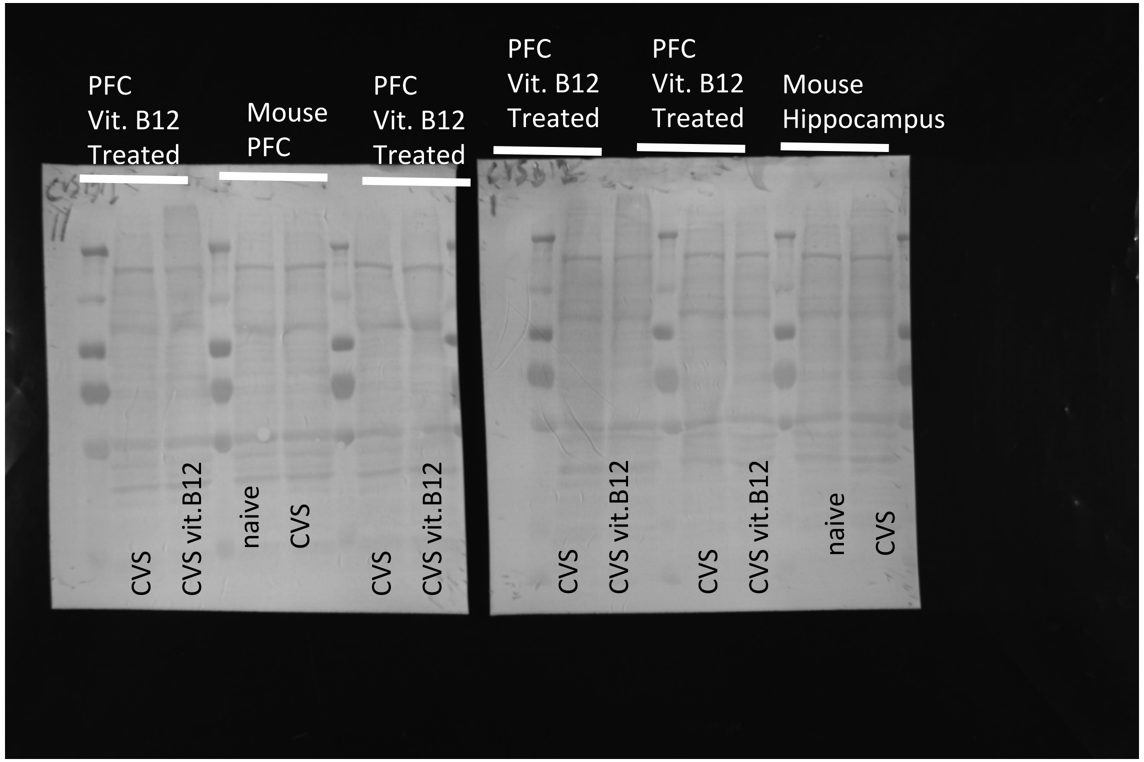

GMPPB

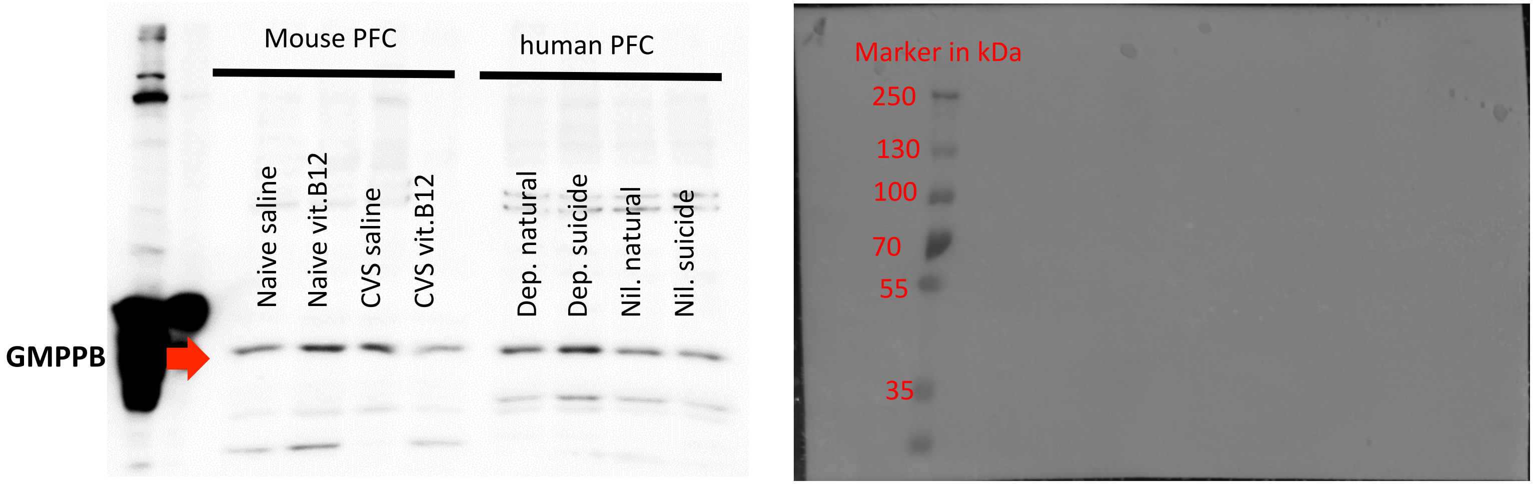
Mouse PFC
human PFC
Naive saline
Naive vit.B12
CVS saline
CVS vit.B12
Dep. natural
Dep. suicide
Nil. natural
Nil. suicide
GMPPB
Marker in kDa
250
130
100
70
55
35

GMPPA

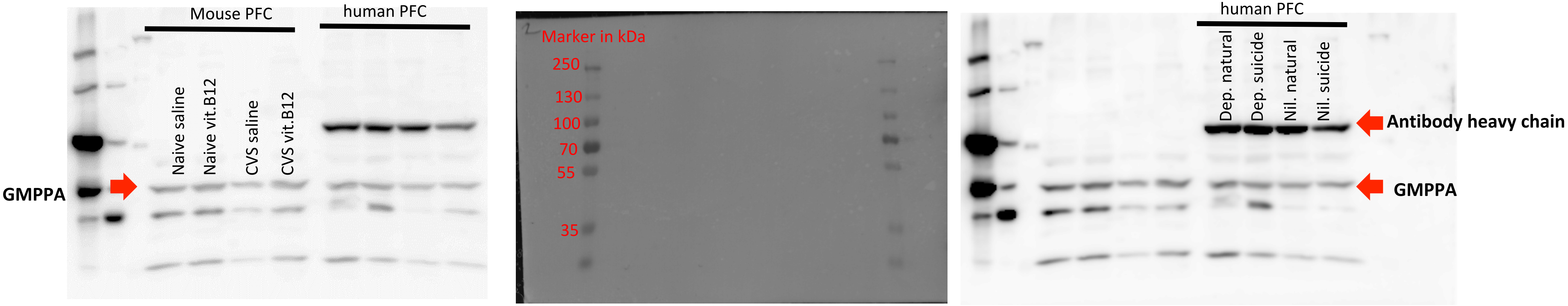
Mouse PFC
human PFC
Naive saline
Naive vit.B12
CVS saline
CVS vit.B12
GMPPA
Marker in kDa
250
130
100
70
55
35
human PFC
Dep. natural
Dep. suicide
Nil. natural
Nil. suicide
Antibody heavy chain
GMPPA

mannosylation

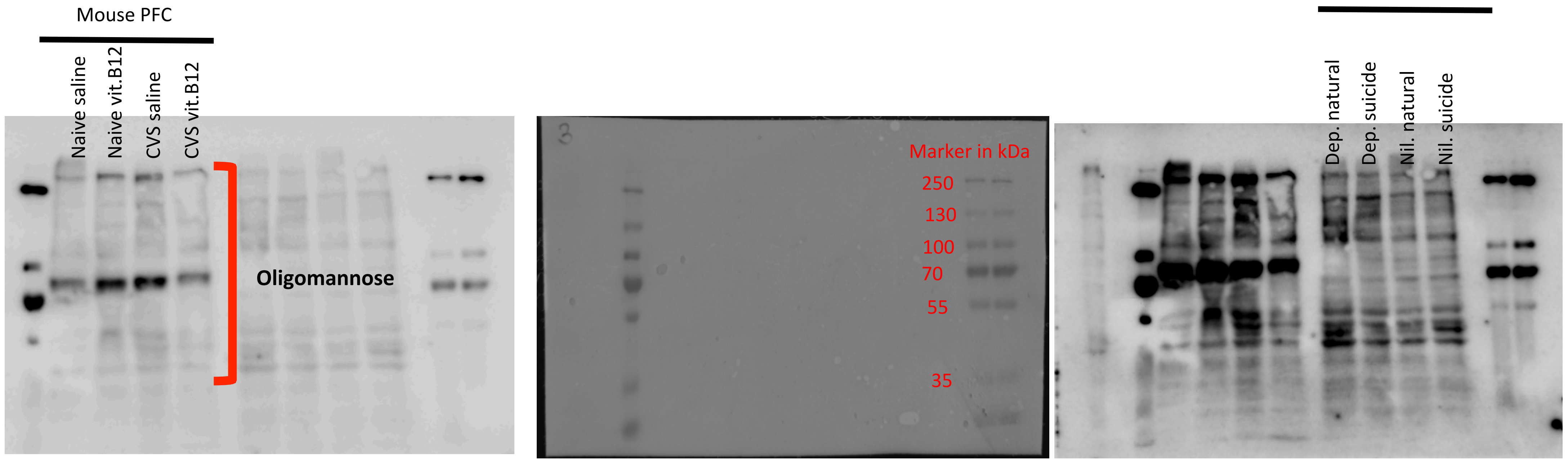

GAPDH (on membrane for mannosylatioon)

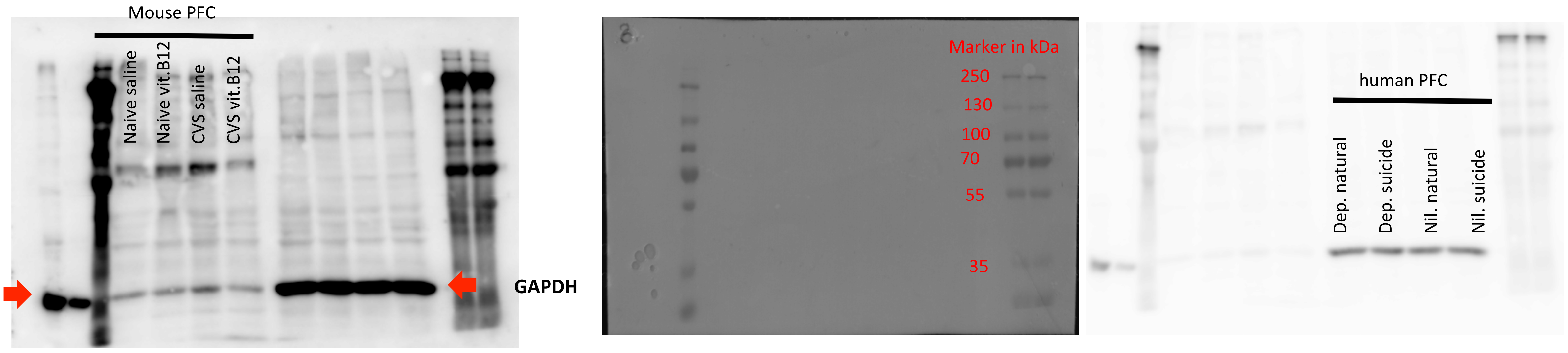

Supplement: Supplementary Figure 5 — Full length western blots. [file Data_Sheet_1.PDF]
